# Supplementary material for: Physiologically relevant acid-sensing ion channel (ASIC) 2a/3 heteromers have a 1:2 stoichiometry
Source: Commun Biol. 2023 Jul 8;6:701. doi: 10.1038/s42003-023-05087-4 (PMC10329638; doi:10.1038/s42003-023-05087-4)
Supplement: Supplementary file 2 — Supplementary Information [file 42003_2023_5087_MOESM2_ESM.pdf]

Supplementary Information for

Physiologically relevant Acid-sensing ion channel (ASIC) 2a/3 heteromers have a 1:2 stoichiometry

Leon Fischer, Axel Schmidt, Anke Dopychai, Sylvia Joussen, Niko Joeres, Adrienne Oslender-Bujotzek, Günther Schmalzing, Stefan Gründer *

* Corresponding author: Stefan Gründer; Email: sgruender@ukaachen.de

This PDF file includes

Supplementary Figures 1-3

Supplementary Table 1

Supplementary Figures

Supplementary Figure 1. Visualization of ASIC2a-3 concatemers in plasma membrane-bound form and as ^{35}S-labeled total protein.

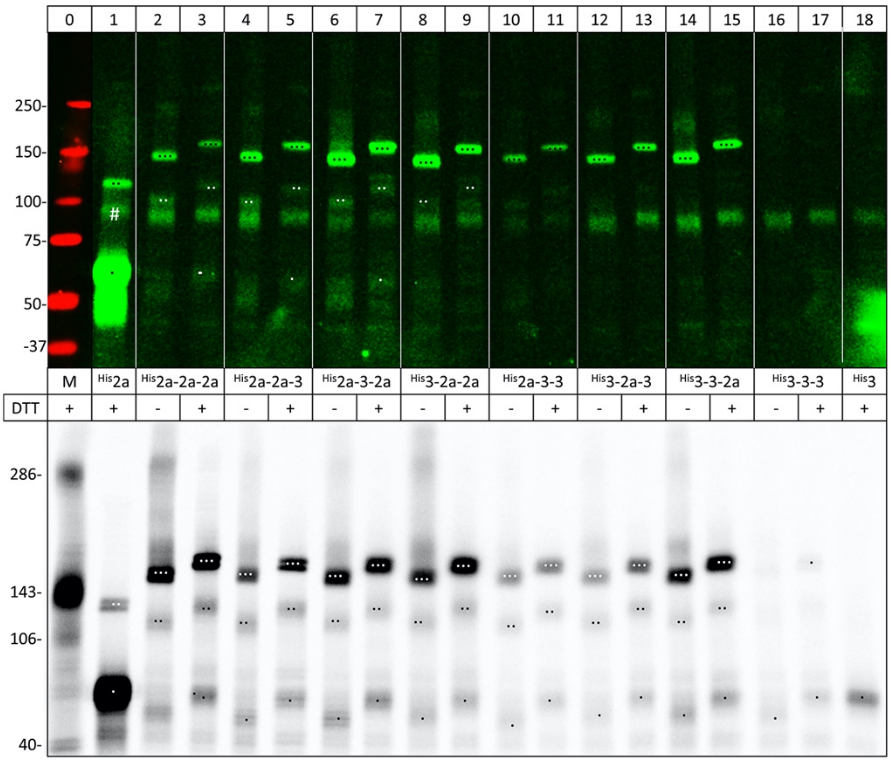

The sequence of numbers 2a and 3 indicates the order from the N to the C terminal end in which the subunits ASIC2a and ASIC3 are concatenated together, respectively. The indicated ASIC proteins labeled with [^{35}S]methionine and IR800 were purified via their heptahistidine tags and resolved by SDS-urea-PAGE. The top and bottom panels are IR800 and ^{35}S scans, respectively, visualizing the ASIC proteins in their plasma membrane bound and total (intracellular plus plasma membrane-bound) forms. Lane 0 shows the migration of the Precision Plus Protein All Blue protein standard (BioRad, top panel) and the three oocyte-expressed, ^{35}S-methionine-labeled human proteins (bottom panel) His-rapsyn (40 kDa), His-TRPC6 (106 KDa) and His-TMEM16A-GFP (143 kDa); the 286 kDa band represents homodimeric His-TMEM16A-GFP. The number of black or white dots in the protein bands indicates whether they are intact trimers (three dots) or by-products such as dimers (two dots) or monomers (1 dot). #, Background band originating from an endogenous ~90 kDa membrane-bound oocyte protein isolated by the Ni NTA resin.

Supplementary Figure 2. Scheme illustrating how concatemers were generated.

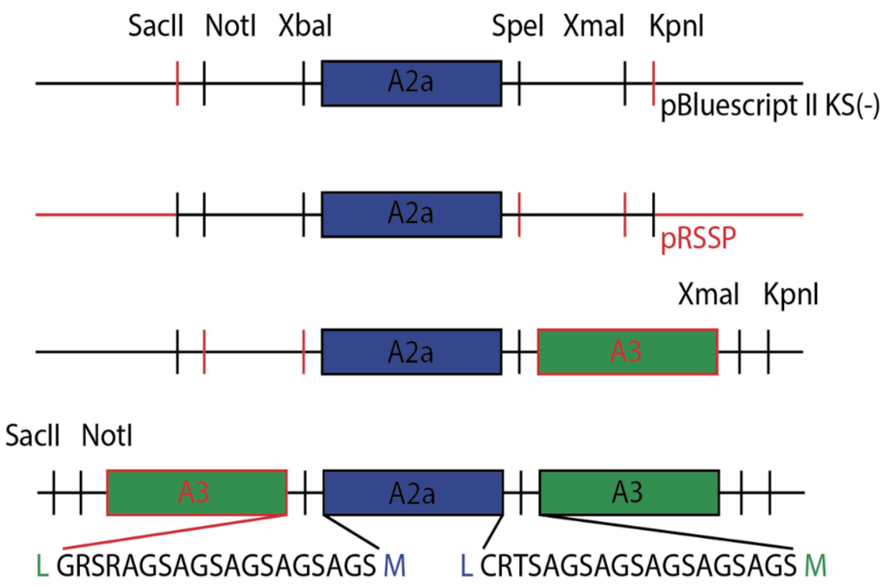

Succesive steps are shown from top to bottom. Restriction sites that were used for the next subcloning step as well as changes that have taken place are highlighted in red. In this scheme, the generation of the concatemer ASIC3-ASIC2a-ASIC3 is shown, but the same steps were performed for all concatemers used in this study. The linker-sequences are given in the single-letter code for amino acids. Note that the stop-codons of the subunits at position 1 and 2 were removed. SacII, NotI, XbaI, SpeI, XmaI, and Kpn: restriction sites. pBluescript II KS (-) and pRSSP: plasmids. A2a: rat ASIC2a, A3: rat ASIC3.

Supplementary Figure 3. Unprocessed scans of the gel, used to generate Supplementary figure 1.

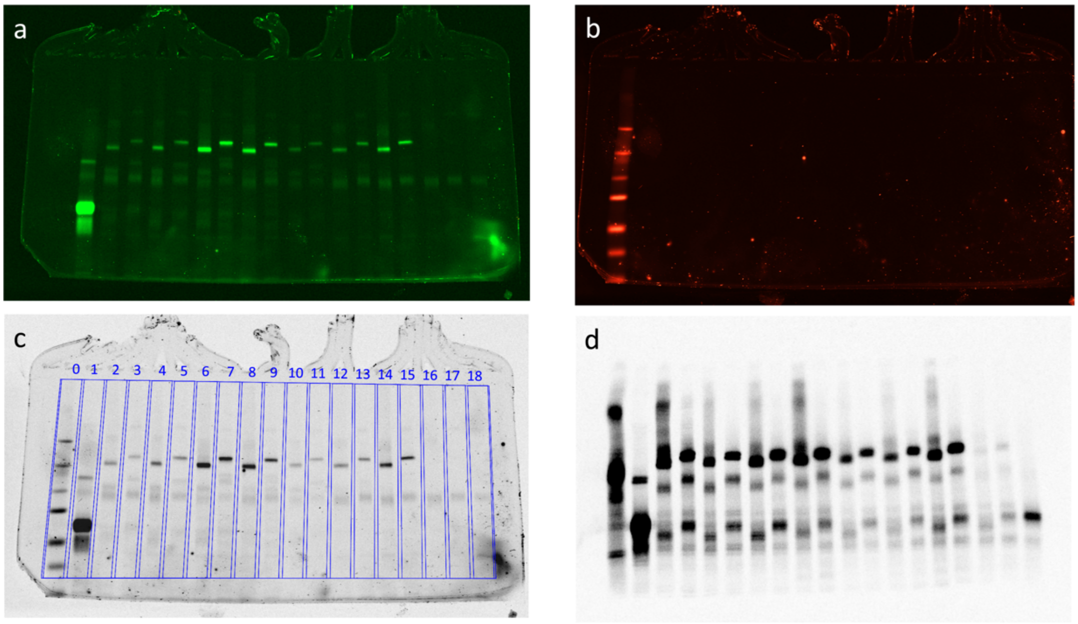

Original unprocessed scans of an entire SDS-urea-PAGE gel of freshly purified ASIC proteins. All lanes of the SDS-urea-PAGE gel are included in Supplementary figure 1. Separate scans were required due to different detection principles: protein-bound IR800 fluorescence and BioRad Precision Plus Protein™ standards were detected with a LI-COR Odyssey scanner at 800 nm and 700 nm, respectively, and proteins with metabolically incorporated ^{35}S were detected with a PhosphorImager (Storm 820, GE Healthcare). Supplementary figure 1 is a combination of the scans. Panels a, b, and d show IR800 fluorescence of plasma membrane-bound proteins (a), BioRad protein standards (b), and metabolically ^{35}S-labeled proteins (d). Panel c illustrates the lane numbering as used in Supplementary figure 1. The ^{35}S mass markers shown and specified in Supplementary figure 1 have been omitted from lane 0 in panel c, but are visible as the outer left lane in d.

Supplementary Table

Supplementary Table 1: Electrophysiological properties of ASIC1a and ASIC1a concatemers.

		pH_{50} (Act)	Hill (Act)	n	I_{max} (μA)	n
ASIC1a	WT	6.59 ± 0.02	3.18 ± 0.41	10	32.71 ± 3.87	9
Concatemers	WT-WT-WT	6.45 ± 0.02	2.38 ± 0.3	15	11.89 ± 2.16	7
	HN-WT-WT	6.51 ± 0.02	2.72 ± 0.24	24	0.98 ± 0.16	8
	WT-HN-WT	6.08 ± 0.05	1.03 ± 0.07	16	0.75 ± 0.11	7
	WT-WT-HN	5.96 ± 0.05	0.83 ± 0.04	15	0.92 ± 0.27	7
	HN-HN-WT	-	-	-	0.05 ± 0.01	11
	HN-WT-HN	-	-	-	0.22 ± 0.06	8
	WT-HN-HN	-	-	-	0.16 ± 0.07	8
	HN-HN-HN	-	-	-	0.03 ± 0.01	6

Note that this table contains data from two experiments. pH_{50}-values and Hill-coefficients are from concentration-response experiments (see text); values for I_{max} are from current amplitude experiments injecting equal amounts of mRNA.
